# Psychological Impacts of the COVID-19 Pandemic on Rural Physicians in Ontario: A Qualitative Study

**DOI:** 10.3390/healthcare10030455

**Published:** 2022-02-28

**Authors:** Anchaleena Mandal, Eva Purkey

**Affiliations:** 1 School of Medicine, Queen’s University, Kingston, ON K7L 3N6, Canada; eva.purkey@queensu.ca; 2 Department of Family Medicine, Queen’s University, Kingston, ON K7L 3G2, Canada

**Keywords:** COVID-19, mental health, psychological impact, pandemic, epidemic, primary care physicians, coronavirus disease, rural

## Abstract

Frontline rural physicians in Canada are vulnerable to the psychological impacts of the COVID-19 pandemic considering their high pre-pandemic burnout rates as compared to their urban counterparts. This study aims to understand the psychological impacts of the COVID-19 pandemic on rural family physicians engaged in full-time primary care practice in Ontario and the stressors behind any identified challenges. Recruitment combined purposive, convenience, and snowball sampling. Twenty-five rural physicians participated in this study. Participants completed a questionnaire containing Patient Health Questionnaire-2 (depression), General Anxiety Disorder-2 (anxiety), and Perceived Stress Scale-4 (stress) screening as well as questions exploring self-reported perceptions of change in their mental health, followed by a semi-structured virtual interview. Quantitative data showed an overall increase in self-reported depression, anxiety, and stress levels. Thematic analysis revealed seven qualitative themes including the positive and negative psychological impacts on rural physicians, as well as the effects of increased workload, infection risk, limited resources, and strained personal relationships on the mental health of rural physicians. Coping techniques and experiences with physician wellness resources were also discussed. Recommendations include establishing a rapid locum supply system, ensuring rural representation at decision-making tables, and taking an organizational approach to support the mental health of rural physicians.

## 1. Introduction

The Coronavirus Disease 2019 (COVID-19) pandemic has introduced additional burdens on the publicly funded Canadian healthcare system, alongside great uncertainty and personal health risk for people working within the healthcare system. This has resulted in significant psychological impacts on frontline healthcare workers (HCW), who experience high levels of work-related stress even under normal circumstances [1]. This is particularly true for rural and remote communities, comprising approximately 21% of the Canadian population, which are primarily serviced by general practitioners [2,3,4]. While one in four Canadian physicians and residents reported high levels of burnout before the pandemic, the available pre-pandemic literature also shows profoundly reduced wellness, increased burnout, decreased job satisfaction, and lower retention rates of rural physicians as compared to their urban counterparts [3,4,5]. This can be attributed to limited resources, longer work hours, reduced access to specialty consultation, lack of privacy from patients outside of the clinical context, difficulty in taking time off, and increased social isolation among other factors [3,4,6].

Global research during the COVID-19 pandemic and the previous SARS pandemic shows higher rates of depression, anxiety, fear of infection, stress, burnout, isolation, poor sleep quality, exhaustion, psychiatric disorders, and even suicide of HCWs, including physicians [7,8,9,10,11,12,13,14,15,16,17,18,19,20]. The current international literature on rural HCWs shows similar worsening mental health during the COVID-19 pandemic [11,21,22]. The consequences of excessive and prolonged stressors can be detrimental to the quality of care provided, resulting in reduced patient satisfaction, compassion fatigue, lower productivity, absences, and higher turnover rates [23,24]. Increased rates of medical errors, breach of protocols and guidelines, and patient safety concerns are also a risk [23,24]. Supporting the mental health of physicians is, therefore, also vital for sustaining an effective response from the medical workforce during the pandemic, particularly in underserviced rural areas.

In October 2020, the Canadian Medical Association (CMA) responded to the mental health crisis of physicians by introducing a Physician Wellness Initiative [25]. Other organizations, such as the Society of Rural Physicians of Canada (SRPC) and Canadian Medical Protective Association (CMPA), posted lists of online COVID-19 and mental health resources [26,27]. Some hospitals have developed “grief circles” and response teams to provide urgent support to distressed physicians [28,29]. Nevertheless, in February 2021, a survey by the CMA showed that 64% of respondents were still experiencing anxiety and reported that their fatigue had increased by 69% within the first year of the pandemic [30]. This may be because most of these services do not address the underlying challenges, and physicians may be reluctant to disclose mental health distress due to the fear of stigma [1]. Furthermore, there are little to no physician wellness services that specifically address the unique psychological challenges of rural doctors. There is also a paucity of pre-pandemic research on rural physician wellness in Canada. Therefore, this paper will examine the psychological impacts of the COVID-19 pandemic on rural physicians from the province of Ontario within Canada and the stressors behind any identified challenges.

## 2. Methods

### 2.1. Participants

This study recruited participants from the province of Ontario, which had the highest total number of COVID-19 cases in Canada at the time, the fewest hospital beds per capita, a severe shortage of staff, and a greater number of rural residents than any of the other provinces [7,31,32]. This study uses Statistics Canada’s definition of rural, which is defined as communities with less than 10,000 people and are located outside of the commuting zones of larger urban centers [33].

Recruitment combined purposive, convenience, and snowball sampling between May and early August 2021. A recruitment poster and letter were advertised through the SRPC, on social media, and by contacting rural hospitals and affiliated rural sites of medical schools in Ontario. The inclusion criteria included physicians with College of Family Physicians of Canada certification, working in rural Ontario since at least March 2020, and having a full-time primary care practice with regular duties in at least two clinical settings (i.e., emergency department, clinics, hospitals, long-term care, etc.). Participants were recruited to the point of thematic saturation, when data collection no longer resulted in the identification of new themes [34]. This was confirmed when researchers started to hear the same comments again and again, and there were no new codes. Ethics approval was obtained through the Queen’s University Health Sciences and Affiliated Teaching Hospitals Research Ethics Board. Participants were provided a $50 SRPC gift card honorarium.

### 2.2. Data Collection and Analysis

Once participants were verified as fitting the inclusion criteria, they were emailed a brief online questionnaire, which collected demographic information, a Generalized Anxiety Disorder (GAD)-2 score, a Patient Health Questionnaire (PHQ)-2 score, a Perceived Stress Scale (PSS)-4 score, and self-reported perceptions of change in their mental health compared to before the pandemic [35,36,37]. The purpose of collecting these quantitative descriptive data was to understand the current mental health status of the participants. GAD-2 is a two-item tool with a cut-off at ≥3 points that rapidly screens for Generalized Anxiety Disorder (sensitivity 76%, specificity 81%) [35]. Similarly, the PHQ-2 is a 2-item screening tool for the presence of depression (sensitivity 97%, specificity 67%) with a cut-off at ≥3 points [36]. Finally, the 4-item PSS-4 is the most widely used psychological instrument for measuring the perception of stress and has an internal reliability of r = 0.60 [37].

The questionnaire was followed by an approximately 60-min one-on-one semi-structured interview over Zoom video conferencing or phone call. The interview guide included seven questions and associated prompts that were developed through an extensive review of the literature on the topic of interest. Please see Appendix A Interview Guide for a copy of the interview guide.

The qualitative data of this study was analyzed using thematic analysis, which allows for the identification of recurring ideas (referred to as themes) in a data set [38,39]. Data analysis was conducted by the primary researcher (AM). Interviews were audio-recorded and transcribed verbatim. Interview transcripts were imported into NVivo12 (QSR International, Burlington, MA, USA). All data were read in its entirety, encoded into twenty-four codes, and key conceptual themes were searched, reviewed, and defined by AM. Themes and codes were discussed iteratively with the research supervisor (EP) to ensure validity. An external family physician in rural Ontario was consulted regarding the research findings and recommendations to check for relevance and sensitivity to the rural physician community.

## 3. Results

### 3.1. Quantitative Findings: Demographic and Questionnaire Results 

Twenty-five rural physicians participated in this study. Please reference Table 1, Table 2 and Table 3. Most participants were between the ages of 35 and 49 years. There was a relatively equal distribution between participants from Southern and Northern Ontario as well as between male and female gender, with one participant who identified as trans, gender fluid, or non-binary. The majority of participants (72%) scored between 6 and 10 on the PSS-4 screening, indicating moderate stress levels. Although 16% screened positive for depression in PHQ-2 and 44% screened positive for anxiety in GAD-2 at the time of the study, the majority of the participants self-reported increased levels of perceived depression (48%), anxiety (72%), and stress (92%), as well as a decreased sense of overall mental wellbeing (80%) compared to before the pandemic. Full results of PHQ-2, GAD-2, and PSS-4 are reported in Table A1.

### 3.2. Qualitative Findings

**Figure 1:**
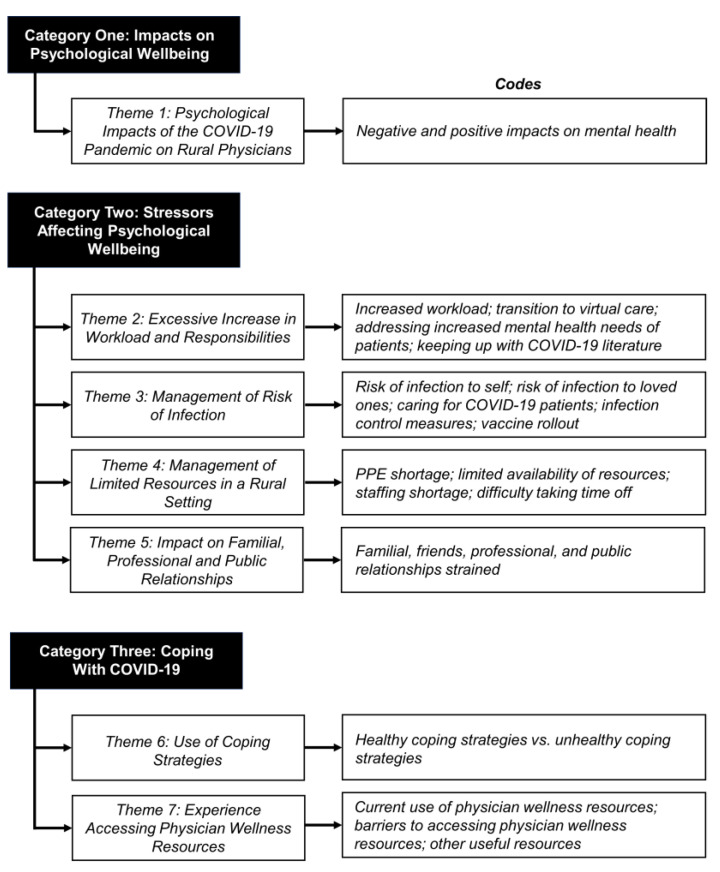
Qualitative thematic analysis with categories, themes, and associated codes.

From the qualitative data, seven main themes emerged (Figure 1) that are organized into three broad categories. Please refer to Table A2, Table A3 and Table A4 for quotes relevant to each theme.

#### 3.2.1. Category One: Impacts on Psychological Wellbeing

##### Theme 1: Psychological Impacts of the COVID-19 Pandemic on Rural Physicians

Although rural Ontario generally had delayed onset and a low number of COVID-19 cases due to geographical isolation and eventual high vaccination rates, participants described how the trickle-down effects on the rural healthcare system continued to impact the psychological wellbeing of most rural physicians to varying degrees. Almost all participants reported that their mental health had worsened at the beginning of the pandemic. However, over time, as they learned to adapt, their mental health either stayed the same or seemed to improve, although still at an overall lower level than their baseline from before the pandemic.

Beyond work, many physicians struggled to balance family obligations, social isolation, and personal struggles such as loss of loved ones, going through a divorce, managing personal/family member health issues, and worrying about loved ones in long-term care homes. Common words used by participants to describe their mental health included frustration, anxiety, stress, tired, anger, irritable, hopeless, drowning, defensive, burnout, COVID-fatigue, depression, disappointed, emotional, low mood, and numb.

Earlier in the pandemic, anxiety largely stemmed from the uncertainty surrounding the virus. Many local public health projections predicted massive outbreaks, which generated fear of the worst-case scenarios and “feeling on edge” in understaffed and low resourced rural centers. This anxiety manifested as “doom and gloom” conversations, feeling behind on scientific readings, feeling underprepared for work, as well as worrying about long-term impacts on personal life, hospital backlog, the economy, etc.

Outbreaks in rural communities triggered anxiety, a sense of demoralization, and feelings of helplessness, and several participants found it stressful to deal with the medical complications of decompensating COVID-19 patients in a low-resource setting. Some participants also resented that public health protocols for COVID-19 patient care undermined the dignity of the patient and restricted visits from family during end-of-life care. The death of a COVID-19 patient was personally difficult for many rural physicians, as individuals in rural communities are usually intimately familiar with each other, and physicians know them as more than just patients.

As the pandemic progressed, feelings of burnout evoked self-questioning about the purpose of working when they were unable to enjoy their personal lives, especially in younger physicians who may place higher value on work–life balance. They reported feeling like there was no end in sight and not having a life outside of work. Interestingly, previous experience of the SARS pandemic appeared to have helped older physicians to mentally prepare themselves for the COVID-19 pandemic.

A few participants identified an overwhelming feeling of constant generalized anger at the beginning of the pandemic relating to their stressful workload and dissatisfaction with the government, public reaction, and vaccine rollout. This often manifested as easily losing patience with uncooperative patients, emotional outbursts, and strained relationships with colleagues and family.

Interestingly, despite adverse psychological impacts, some participants also felt that it was exciting to deal with a new challenge through teamwork with their colleagues and rewarding to be able to use their skills to help their community. This sparked feelings of “rising to the challenge” and “humble gratitude.”

#### 3.2.2. Category Two: Stressors Affecting Psychological Wellbeing

##### Theme 2: Excessive Increase in Workload and Responsibilities

Planning and preparations for the COVID-19 pandemic, such as setting up assessment centers, restructuring teams, practicing protected code blue simulations, modifying ventilator capacity, etc., were a substantial source of stress for rural family physicians. This is because, unlike urban centers, rural generalists work across multiple practice settings and, thus, were responsible for enforcing COVID-19 protocols across several departments. Participants described it as “scrambling” to adjust to the new practice and protocols, and many physicians returned early from their vacation to assist.

Initially, clinic and emergency department workload decreased as patients delayed accessing healthcare due to fear of contracting COVID-19, but the workload quickly spiked as patients started coming with acute presentations requiring a higher level of care. The creation of new roles in the assessment center, vaccination clinics, and additional services added strain on the already understaffed system. Physicians now also had to cover for their colleagues on short notice while the colleague was awaiting swab results or recovering from illness. Workload and stress further increased during peaks of COVID-19 outbreaks, and the transfer of complex or ventilated patients to overwhelmed tertiary centers was difficult to organize. Many participants reported greater anxiety because of the unpredictability of their workload with the changing nature of the pandemic.

With the transition to virtual care, physicians were now taking on more patients per day and, thus, having to do more paperwork. Their workload also substantially increased as they were seeing some patients twice, virtually followed by in-person. Concerns of the physicians included doing more investigations to compensate for the lack of a physical exam, fearing that they were providing inferior quality of care virtually, and frustration dealing with patients who were resistant to this change. However, one participant reported enjoying virtual care as they were able to work from the comfort of their home.

Most participants expressed empathy for the increased mental health challenges and struggles of patients during the pandemic for reasons such as unemployment, isolation, difficulty finding childcare, etc. In response, some clinics extended their appointment duration to accommodate mental health discussions. However, some participants disclosed that having to deal with so many mental health appointments contributed to second-hand anxiety, compassion fatigue, and/or exhaustion.

The administrative workload also increased, with more communications, emails, paperwork, as well as investigation results to review. Moreover, there was a drastic increase in the number of meetings for COVID-19-related preparations and decision-making or policy making, in turn adding multiple hours to their workweek.

All participants reported feeling a sense of duty to keep up with the rapidly evolving COVID-19 literature, protocols, and guidelines to provide evidence-based patient care or to develop policies for their local hospital, often spending hours and working late at night to catch up. The majority expressed feeling information overload, cognitive fatigue, and exhaustion over time. Differing and conflicting messaging from various sources and lack of relevance to the rural context created confusion, prompting some participants to selectively review a few reliable information sources or to only consume the literature that was practical for their daily use. Frustration was also augmented by political influence on public health decisions. It was especially stressful for physicians who were responsible for translating the evolving literature to the public on behalf of their hospital. As information rapidly changed, most notably during the AstraZeneca vaccination controversy, many physicians felt that the public lost confidence in the guidance of physicians, and many physicians in turn lost trust in government messaging, which one participant described as “shady” and “lacking transparency” [40].

Out of professional responsibility, commitment to their community drove rural physicians to take on more responsibilities and workload. With time, this led to burnout, and some physicians gave up leadership positions and refused more work. New grads reported burning out faster and feeling under-skilled. On the other hand, provincial restrictions eventually made travel more difficult for locum physicians, so some of them chose to either spend longer periods of time working in a community or to settle down in a permanent practice. Because they were no longer able to locum for short periods and take immediate breaks, taking on full or longer practice increased their workload and fatigue. Locum physicians who continued to travel expressed frustration with multiple quarantines, swab testing, and differences in regional protocols.

##### Theme 3: Management of Risk of Infection

In general, younger physicians were less anxious about personal infection risk compared to older physicians, who felt more vulnerable and some of whom feared death due to their older age and co-morbidities. Personal fear stemmed from insufficient knowledge about the virus, not having adequate personal protective equipment (PPE), and lower intubation proficiency among other factors. Physicians with young children were particularly anxious about what would happen to their children if they died from COVID-19. Some physicians reported that they had reviewed their will, revised advanced directives, and shared account passwords with their partner in apprehension of their death. Nevertheless, most participants reported feeling desensitized to the risk of infection over time, and vaccines provided them with a greater sense of safety.

Many participants feared bringing home COVID-19 to their family or spreading it to vulnerable patients. A few participants even experienced stigma from family members. As a result, some physicians reduced their contacts, temporarily lived separately from their family, avoided entering nursing homes unless necessary, strictly adhered to infection control and PPE protocols, wore scrubs at work, and/or developed a sanitization ritual after coming home from work (i.e., washing clothes and showering before interacting with family). Several participants reported choosing to stay separate from their family during self-isolation while awaiting swab results, which was emotionally difficult but relatively easy to do in a larger rural home.

Participants who had a greater sense of personal risk of infection also expressed frustration with individuals who did not strictly follow COVID-19 protocols, demonstrated negligence, or had a lower perception of infection risk. Some physicians reported feeling a responsibility to gently remind colleagues and patients to wear masks (like a “PPE police”) and worrying that other staff may become lax with protocols over time.

Most COVID-19 patients were managed at home or transferred to tertiary care center intensive care units (ICU) due to lack of infrastructure to care for complex conditions in rural centers. Many participants did not know that they had cared for a COVID-19 patient until later when swab results returned. These participants often reported more post-exposure anxiety due to fear of not having taken enough precautions at the time. Participants also reported that as rural generalists are not as experienced with intubating as anesthesiologists in urban centers, they felt greater anxiety with intubations and other aerosol generating procedures that would increase the risk of infection for healthcare providers.

Infection control measures and cleaning protocols fostered an increased sense of safety. However, after a while, the majority of participants found these protocols to be an additional annoyance, such as having to screen patients, skin irritations from cleaning products, putting on full PPE before seeing every patient, waiting for rooms to dry after sanitization, not being able to eat at their desk or in a break room, as well as entering and exiting through different doors. Protected code-blue barriers also made it more difficult to manipulate equipment and provide quality care.

Although healthcare professionals were labelled as the priority during the COVID-19 vaccine rollout, most participants reported that the general public in several urban centers were receiving the vaccine before them. Rural physicians felt that their risk of infection was “ignored” and felt insulted and undervalued by not being prioritized for vaccines despite the vulnerability of short-staffed rural centers. They were also frustrated by the variation in rollout between communities, the redirection of vaccines away from rural communities, or receiving an insufficient vaccine supply for their population. Several participants reported feeling frustrated by the lack of cooperation of health units to plan for vaccine clinics and later being instructed to set-up a vaccine clinic at the last minute.

##### Theme 4: Management of Limited Resources in a Rural Setting

The global shortage of PPE at the beginning of the pandemic also impacted unprotected frontline rural physicians, causing much stress and anxiety. Some participants felt neglected by local integrated health networks (LIHN), which are regional organizations responsible for planning, integrating, and funding local health services in Ontario, as they did not provide PPE for community physicians [41]. At some rural hospitals, there was also conflict over the banned use of personally supplied masks at the beginning of the pandemic. To mitigate the PPE shortage issue, many rural centers collected donations from the community and sub-optimal handmade PPE by local sewers, retrieved expired PPE stock from past Ebola, H1N1, and SARS precautions, and reused PPE when possible. No participants reported running out of PPE, but most reported having to use their reserve sparingly until supplies improved.

Use of PPE also had its negatives such as mask acne, headaches from visors, visors steaming up, being dehydrated, and sweating in PPE, especially during hot summers. PPE also made it more difficult to communicate with patients who lip read or are hard of hearing and decreased the human touch in patient care. Nevertheless, most participants did not mind PPE as part of their job and felt safe and grateful to have it.

The limited COVID-19 swab testing capacity and long result turnaround times earlier in the pandemic disproportionately affected short-staffed rural communities where they were burning through PPE and losing potentially exposed staff while awaiting test results. Unprotected frontline physicians also felt unsafe not knowing if their patient had COVID-19 due to limited testing capacity. Moreover, many rural hospitals have access to only one or two transport ventilators and limited oxygen supply, so participants were fearful of running out of these resources if they were unable to transfer ventilated patients out to overwhelmed tertiary care centers during a local COVID-19 outbreak. This worry was compounded by the limited training of rural nurses to care for complex ventilated patients. Interestingly, limited resources prompted many rural physicians to focus more on the patient’s goals of care, such as providing palliative care at home as per the patient’s request instead of seeking aggressive care in a tertiary care center ICU where they might die alone or contract COVID-19 if ill with something else.

On top of the chronic shortage of human resources, the creation of new roles and clinics during the pandemic required rural family physicians to take on extra shifts, cover for quarantining colleagues, work “double duty” (cover two services concurrently), and be deployed to ICUs with minimal training. Many older physicians were unable to retire as they could not find someone to take over the care of their patients. Moreover, taking time off was difficult as there was a profound shortage of locum physicians, and permanent physicians felt guilty for creating additional burden on colleagues to cover them. As a shortage of staffing threatened some rural emergency departments to close, physicians felt an obligation to continue working to ensure continuity of healthcare services in their community. Even if they were able to take time off, their break was fragmented by being called in to work extra shifts, completing paperwork, and other duties. In other words, there is a culture in rural centers to constantly be on-call if within town, even during time off. Participants explained that true time off requires leaving town, which was not possible due to COVID-19 travel restrictions. Similarly, taking time off did not feel worthwhile for some, as they were unable to visit loved ones in other communities.

Over time, overworked rural physicians started feeling the effects of burnout. The provincial government provided funding to incentivize rural physicians to continue working and keep emergency departments open, but almost all participants revealed that they would prefer more time off to care for their physical and mental health needs rather than earning more money.

##### Theme 5: Impact on Familial, Professional, and Public Relationships

The social life of rural physicians became limited during the pandemic due to public health restrictions. At work, socializing opportunities such as eating and sharing food with colleagues were halted. Virtual care and decreased face-to-face time with patients and colleagues caused increased professional isolation. Participants who were locums reported that travel restrictions prompted some of them to settle in a community, where they struggled to develop a new social circle due to social restrictions. This enhanced their sense of isolation as their main social supports were residing in distant locations. One participant even contemplated terminating their contract to be with their loved ones. Physicians who were single, unable to date, and living alone also reported feeling a great sense of isolation. However, at the same time, participants reported feeling an increased sense of camaraderie with their rural colleagues as they worked together through the various challenges presented by the pandemic.

The impact on familial relationships varied. Most participants reported that their partner was very supportive, and that having a partner who took care of home demands was helpful. Common regrets included not spending enough time with their partner, being cranky, and feeling like their partner did not understand a physician’s stress. Rural physician couples or couples with both individuals working in healthcare struggled with being too busy with work, having differing views about the pandemic, talking about COVID-19 all the time, or staying separate from each other so that the hospital did not lose two doctors to illness at the same time. Virtual schooling or homeschooling allowed physician parents to spend more time with their kids at home, although some participants regretted being too busy to see their child’s growth or being irritable with their children. Physicians with young children reported more burnout as they were either working or they were parenting at home, with no time to care for their own needs. Other common concerns included difficulty finding childcare, worrying about children’s mental health and safety, and worrying about the quality of virtual education.

Most rural physicians limited in-person interactions with extended family and friends due to pandemic restrictions and to protect elderly/vulnerable family members. While most stayed connected virtually, some met with friends in outdoor settings such as on hikes, picnics, or sitting around a fire. A few participants expressed being too busy to stay connected, or they avoided contact to limit COVID-19-related discussions, or the frustration of their work stresses not being understood by others. Participants also reported feeling judgmental towards those who did not follow public health guidelines, chose not to get vaccinated, or did not share the same sense of risk of infection or view about the pandemic as them.

At the beginning of the pandemic, most rural physicians reported feeling grateful and motivated by the appreciation from the public for being “healthcare heroes.” However, a few participants felt it was misplaced since it was their job and other essential workers, such as grocery store employees, deserved more appreciation. One participant also reported feeling less like a hero and more like “cannon fodder” earlier in the pandemic and would have preferred to receive proper PPE and supports instead of the hero label. Later, participants reported feeling like scapegoats for the public’s exhaustion with the pandemic, manifested as increased rudeness, frustration, and lack of cooperation from patients. Rural physicians also felt disheartened by anti-vaccination discussions, anti-masking protests, and patients trusting “internet doctors” over their own evidence-based recommendations. Most physicians tried to avoid social media; people were always asking for their opinion, social media attacks in rural communities are often personal where HCWs are unable to defend themselves, and differences in opinion about the pandemic often shattered relationships.

#### 3.2.3. Category Three: Coping with COVID-19

##### Theme 6: Use of Coping Strategies

The long-term nature of the COVID-19 pandemic posed a challenge for rural physicians to adapt and use their usual healthy coping strategies. Due to restrictions and closures, leisure travel and most recreational activities providing a distraction from work were no longer an option.

While rural physicians initially struggled to cope, the majority of the participants of this study eventually learned to develop healthy coping techniques to retain some consistency in life during their limited free time. One common method was to find ways to distract oneself from work such as limiting COVID-19-related discussions and consumption of news, gardening, practicing music, watching movies, reading books, and doing outdoor activities. Some physicians engaged in activities that increased their mindfulness, positivity, and self-confidence. This included meditation, journaling, self-reflection, therapeutic letter writing, focusing on things one can change rather than things one cannot, celebrating “small wins” from one’s efforts, and educating oneself about COVID-19. Many participants also worked on strategies to improve self-care, balance relationships, and follow a healthy lifestyle such as by exercising, having a reasonable sleep schedule, connecting with loved ones, learning to incorporate breaks, not doing work outside of business hours, and relying on their partner to identify limits. When possible, some participants visited their social supports in other communities to recharge their mental health.

Individuals with unhealthy coping strategies often reported de-stressing by crying, being too busy or exhausted to apply the usual coping strategies, forcing themselves to continue functioning for their loved ones, and repressing their emotions. These individuals were also more likely to have a worsening diet, do less exercise, feel too tired to do activities that they enjoyed, or lose interest in pursuing their hobbies. Most participants reported that their sleep schedule and quality was impacted by more night shifts, staying up late to catch up on paperwork, insomnia from increased anxiety (both generalized and COVID-19-related), and COVID-19-related dreams or nightmares. A few participants also reported drinking more alcohol than usual and using dark humor to reduce their stress.

##### Theme 7: Experience Accessing Physician Wellness Resources

When all participants were asked if they were familiar with or had used any physician wellness resources offered by any professional associations or organizations, only eight participants responded affirmatively. Of them, four individuals reported satisfactory experiences specifically with the Ontario Medical Association’s (OMA) wellness programs and would recommend it to their colleagues, including the OMA Wellness Support Line, positive messages texting pilot project, and the physician leadership support groups.

A major barrier to accessing physician wellness services by rural physicians was reported as being too busy with their increased pandemic workload to read promotional emails on wellness resources, to explore resources online, or to even take time off to focus on their mental health. Participants also felt overwhelmed by the vast quantity of resources and emails from various sources and did not have time to comb through them all. Furthermore, negative past experiences with mental health services deterred many rural physicians from seeking support. For example, six participants reported having tried the OMA wellness program or other help lines in the past but did not find it useful or knew a colleague who received suboptimal support. Five participants expressed wanting supports tailored specifically for rural physicians to avoid urban-based “geographical narcissism” from province-wide programs. Fear of repercussions on their professional license and insurance, as well as fear of stigma from colleagues, family and the community were also described.

Beyond physician wellness services, while some participants felt uncomfortable reaching out to local mental health services due to the lack of anonymity in the rural setting, five participants reported having a personal counsellor or therapist from outside their community. They expressed enjoying the validation and the personalized service they received, including for their non-professional problems. A few participants were hesitant to seek support from colleagues due to the fear of creating an added burden for them, but most participants reported enjoying support from peers and community mentors with whom they had a deeper connection and a shared rural context, compared to a stranger from a help line. Participants cited physician support groups, the SRPC RuralMed listserv, and WhatsApp group chats with colleagues as useful resources during the pandemic.

Almost all the participants opined that despite all of these resources, improving workforce capacity through increased locum coverage or permanent physician staffing was the most important need of rural physicians.

## 4. Discussion

From the literature, it was hypothesized by the researchers that rural physicians would have a high level of emotional distress during the COVID-19 pandemic [7,8,9,10,11,12,13,14,15,16,17,18,19,20,21,22]. Interestingly, PHQ-2, GAD-2, and PSS-4 positive screening rates (Table 2) demonstrated lower levels of distress than the self-reported perceived change in depression, anxiety, and stress levels of the participants compared to their pre-pandemic baseline (Table 3). This can be attributed to the fact that this study was conducted more than a year after the start of the COVID-19 pandemic, but the objective screening tools only looked at the past two weeks to one month. Thus, the questions in Table 3 were a much more sensitive tool for gauging the longitudinal decline in their mental wellness. As discussed in theme 1, most participants agreed that their responses earlier in the pandemic would have reflected greater distress because they learned to cope with this “new normal” over time despite their overall decline in mental health. This reflects the resiliency and adaptability of rural physicians in the face of adversity and the positive impact of adopted healthy coping strategies. However, resilience can only take an individual so far before systemic change is required to resolve their ongoing stressors and prevent long-term psychological harm.

The findings of this study are consistent with the global literature, although the context behind these stressors is different for rural Canadian physicians [7,8,9,10,11,12,13,14,15,16,17,18,19,20,21,22]. Most psychological stressors identified in this study, particularly in theme 2 and 4, stemmed from previously prevailing systemic challenges within the rural healthcare system that were exacerbated by the pandemic [2,3,4].

This study identified increased workload and staffing shortage as the major stressors that prevented participants from finding time for self-care or taking time off during the pandemic. Even before the pandemic, it was difficult to take time off and arrange for coverage by locums or colleagues due to the chronic shortage of physicians [42]. This contributes to burnout, which was further complicated by the fact that physicians in general are more likely to reach out for mental wellness supports when they are in an acute dire crisis rather than earlier [43].

Health Force Ontario, a marketing and recruitment agency under the provincial government, currently offers centralized and coordinated assistance for locum physician placement across the province through Ontario Physician Locum Programs (including the Rural Family Medicine Locum Program, Emergency Department Locum Program and Northern Specialist Locum Program) to which short-staffed hospitals, individual physicians seeking support and interested locums seeking employment can register [44]. While there is adequate locum supply through these programs, most of them are not available on an urgent basis. Moreover, many employers usually do not arrange locum coverage for their staff and the onus is put on the respective physician. For a physician in crisis, however, the multi-step application process may be cumbersome and time-consuming.

Based on the request of the participants, aside from ongoing efforts to increase recruitment and retention of the rural medical workforce, the researchers recommend the establishment of a rapid, easy-to-access locum coverage system to allow acutely struggling rural physicians to take a much-needed break without worrying about finding someone to cover their practice. This time off will also enable them to access mental health supports and attend to their basic physical needs, so that they can return to work in a better mental health state and provide safe patient care. This is in alignment with the literature on the Physician Wellness Hierarchy, which prioritizes attending to a physician’s physical and mental needs first for them to be able to deliver quality patient care [45]. A logistical model such as the Ontario Physician Locum Programs may be considered but with a simplified application process and standby locums who can emergently ensure continuity of primary medical care in the community.

Most of the stressors identified in this study can be addressed by system-level changes. The CMA positions that health care employers have an ethical responsibility to address physician wellness and remove any occupational and personal barriers to positive physician health [46]. There is also evidence that employer support can be a protective factor for physician mental health during pandemics [1]. An organizational approach, where the employer and hospital leadership are empowered to cultivate a healthy professional culture may, therefore, be considered to address stressors faced by rural physicians. This will also ensure that the approach is relevant to the local context of the physician. Further, employers can play a role in countering mental health stigma in the workplace by fostering open communication; challenging unhealthy attitudes, for example, by ensuring vacation time is protected; hosting an educational campaign about the true impacts of seeking mental health support on licensure and insurance; providing options to seek mental health supports locally or external of their community [1]. When physicians perceive that their efforts are recognized and reciprocated by employers and authorities in these ways, their psychological outcomes, burnout rates, motivation, and morale can be significantly improved [47,48,49].

Finally, during the COVID-19 pandemic, many new urban-centered decision-making committees were established on short notice to develop COVID-19 management policies, but rural representation was not always considered on these teams. As articulated by the study participants, this led to many unique rural challenges being unaddressed, including inequities in access to healthcare resources such as PPE, oxygen, ventilators, and vaccines, which caused increased anxiety for rural physicians and threatened the stability of fragile low-staffed rural healthcare centers. Participants also reported feeling ignored and unvalued by urban-based policy makers. While the *Equity and Diversity in Medicine* policy by the CMA recognizes geographical minorities, it is not as commonly applied in practice compared to other minority populations [50]. Urban-based policymakers also often try to implement urban interventions to rural areas without understanding that it may not always be successful in the rural context [51]. If rural physicians are represented at decision-making tables, they can effectively guide discussions and inform decisions on issues relevant to rural communities. To improve rural representation at the provincial and national level, greater advocacy for the recognition of rural as a minority group is warranted, so that future policies and planning will be inclusive of the rural context and the voice of rural physicians is heard.

## 5. Limitations

A major limitation of this study is its small sample size, which may lower the statistical power of the study. As anticipated, it was difficult to recruit participants who had enough time and energy to contribute to this study due to their heavy workload, burnout, and family responsibilities. This may also explain why there was no participants from communities with less than five physicians.

Participant bias may exist in this study as physicians who are distressed or have strong opinions on the topic are more likely to volunteer as a participant. Further, while the demographic distribution of the participants in this study was diverse, there are certainly many physician perspectives from rural Ontario that were missed. As healthcare is under provincial jurisdiction in Canada, the findings of our study may also not be generalizable to the experience of rural physicians in the rest of Canada or to rural physicians in other countries, despite some similarities.

Finally, thematic saturation was not quantitatively assessed in this study due to the challenges of measuring the degree of saturation statistically. Instead, this study determined thematic saturation through the widely accepted empirical approach proposed by Glaser and Strauss (1967) [52]. Despite its popularity, there are a few limitations that are inherent to this approach. It is dependent on the researcher’s subjective judgement to determine when thematic saturation has been achieved. Moreover, an observation that does not contribute new themes cannot necessarily guarantee that a future observation will not contribute new themes [34]. These limitations can impact the validity of the study.

## 6. Conclusions and Implications

The COVID-19 pandemic has introduced immense stresses on the Canadian healthcare system and the mental health of frontline physicians [1]. This is especially significant for physicians living in under-resourced rural and remote areas, who were already experiencing high pre-pandemic burnout rates [3,4]. For physicians working in rural Ontario, the COVID-19 pandemic has resulted in worsening self-reported overall mental wellbeing due to stressors such as increased workload, risk of infection, limited access to resources and staffing, as well as changing personal relationship dynamics. Current physician wellness resources are often not accessed by burnt-out rural physicians due to difficulty in finding time to focus on self-care. This study recommends establishing a rapid locum supply system, ensuring rural representation at decision-making tables, and taking an organizational approach to support the mental health of rural physicians.

The literature on physician wellness in the rural Canadian context was limited prior to the pandemic. The findings of this study can, thus, be used to guide future research and long-term studies on this important topic in other rural regions of Canada or the world. Healthcare policymakers may also use this data for the development of focused strategies that address rural physician wellness and improve the resilience of the medical workforce. This will, consequently, strengthen the quality of care in rural and remote communities and equip the Canadian healthcare system to effectively support rural physicians during the present and future global public health emergencies.

## Figures and Tables

**Table 1:** Demographics of participants.

Variable	Response % (*n* = 25)	Variable	Response % (*n* = 25)
*Age, years*		*No. of physicians in practice*	
18 to 34	20.0 (5)	<5	00.0 (0)
35 to 49	48.0 (12)	5 to 10	40.0 (10)
50 to 64	28.0 (7)	10 to 20	36.0 (9)
65 and older	4.0 (1)	>20	24.0 (6)
*Gender*		*Medical setting of practice*	
Female	52.0 (13)	Clinics	92.0 (23)
Male	44.0 (11)	Emergency	88.0 (22)
Trans/Gender Fluid/Non-binary	4.0 (1)	Hospitalist	96.0 (24)
Other (No comments)		Obstetrics	20.0 (5)
*Location*		Public Health	8.0 (2)
Northern Ontario	44.0 (11)	Anesthesia	00.0 (0)
Southern Ontario	56.0 (14)	Other (Comments include indigenous communities and reserves, home visits, long-term care, palliative care, MAID, surgical assist, and teaching)	
*Years of practice in Ontario*			
1 to 5	24.0 (6)		
5 to 10	20.0 (5)		
10 to 20	40.0 (10)		
>20	16.0 (4)		

**Table 2:** PHQ-2 (depression), GAD-2 (anxiety), and PSS-4 (stress) screening results.

Variable	Response % (*n* = 25)
*PHQ-2*	
Screened positive for depression	16.0 (4)
Screened negative for depression	84.0 (21)
*GAD-2*	
Screened positive for anxiety	44.0 (11)
Screened negative for anxiety	56.0 (14)
*PSS-4* *	
≤5 (low stress)	24.0 (6)
6–10 (moderate stress)	72.0 (18)
≥11 (high stress)	4.00 (1)

* Please note that PSS-4 results were divided into low, moderate, and high stress groups based on data distribution.

**Table 3:** Self-reported perceived change in mental health of participants from before the pandemic compared to the present.

Variable	Response % (*n* = 25)	Variable	Response % (*n* = 25)
*Level of anxiety*		*Level of stress*	
Increased	72.0 (18)	Increased	92.0 (23)
Stayed the same	28.0 (7)	Stayed the same	8.0 (2)
Decreased	00.0 (0)	Decreased	00.0 (0)
Not sure	00.0 (0)	Not sure	00.0 (0)
*Level of depression*		*Level of overall mental wellbeing*	
Increased	48.0 (12)	Improved	4.0 (1)
Stayed the same	48.0 (12)	Stayed the same	16.0 (4)
Decreased	00.0 (0)	Worsened	80.0 (20)
Not sure	4.0 (1)	Not sure	00.0 (0)

## Data Availability

All data are presented in the article.

## Appendix A

**Interview Guide:**

Questions:

(1) When the pandemic started, how did your everyday medical practice and lifestyle change? Are there are any changes unique to your rural context?

Prompts:

- What new measures were put in place in response to the pandemic?
- Did you have access to appropriate types and quantities of PPE?
- How has your workload changed?
- If you needed it, are you able to get time off as easily as before?
- What personal lifestyle changes have you made?

(2) Do you think your mental health state has changed since before the pandemic compared to now? How?
(3) Do you feel like your community was impacted by many/a high number of COVID-19 cases?

If so, please tell me briefly about that.

(4) What are some of the stressors that have impacted your mental health? What are some stressors that are unique to your rural context in your experience?

Prompts:

- How did the risk of infection impact your mental health?
- How did changing information about the virus impact you (both personally and professionally)?
- How did wearing PPE affect you?
- How did all the new infection-control measures affect you?
- Where there any institutional policies that affected your mental health? Were these impacts positive or negative?
- How did quarantining and self-isolation affect you (both personally and professionally)?
- Did you feel more or less isolated than before?
- Was your sleep schedule impacted?
- How did the balance between your career and relationships get impacted?
- How did the public’s changing perception of your profession impact your mental health?
- How did the anxiety or response of your patients to the pandemic affect you?
- How did taking care of COVID-19 patients make you feel?
- How did availability of resources (i.e., ventilators, PPE, testing kits, medications, staffing, etc.) in your rural setting impact you?
- Did the pandemic have a financial impact on you?

(5) How has the vaccine rollout in rural Ontario impacted your mental health and practice?
(6) What are some coping methods or things you do to maintain or boost your mental health during the pandemic? What did you find worked or didn’t work?
(7) There are several supports that have been made available by the Canadian Medical Association, Ontario Medical Association, Society of Rural Physicians of Canada, and other organizations, including local hospitals. This includes support services, help lines, lists of online resources, etc. Have you ever used any of these resources?

Prompts:

- Do you think the supports are well-tailored to rural physicians like yourself?
- If you did use them, did you find them helpful?/If you didn’t use them, what is an ideal resource that you think will help rural physicians during the pandemic?

Debrief:

(1) Is there anything else you would like to share with me today?
(2) Thank you for sharing your experiences with me today. [Stop recording.] How did you find the interview and questionnaire? Do you have any questions?

**Table A1:** PSS-4, PHQ-2 and GAD-2 detailed results.

Variable	Response % (*n* = 25)	Variable	Response % (*n* = 25)
**PSS-4**		**PHQ-2**	
*Question 1: In the last month, how often have you felt that you were unable to control the important things in your life?*		*Question 1: Over the last 2 weeks, how often have you been bothered by little interest or pleasure in doing things?*	
Never	00.0 (0)	Not at all	48.0 (12)
Almost never	16.0 (4)	Several days	44.0 (11)
Sometimes	56.0 (14)	More than half the days	8.0 (2)
Fairly often	24.0 (6)	Nearly every day	00.0 (0)
Very Often	4.0 (1)		
*Question 2: In the last month, how often have you felt confident about your ability to handle your personal problems?*		*Question 2: Over the last 2 weeks, how often have you been bothered by feeling down, depressed, or hopeless?*	
Never	00.0 (0)	Not at all	48.0 (12)
Almost never	4.0 (1)	Several days	36.0 (9)
Sometimes	28.0 (7)	More than half the days	16.0 (4)
Fairly often	48.0 (12)	Nearly every day	00.0 (0)
Very Often	20.0 (5)		
		**GAD-2**	
*Question 3: In the last month, how often have you felt that things were going your way?*		*Question 1: Over the last 2 weeks, how often have you been bothered by feeling nervous, anxious or on edge?*	
Never	00.0 (0)	Not at all	24.0 (6)
Almost never	4.0 (1)	Several days	32.0 (8)
Sometimes	56.0 (14)	More than half the days	40.0 (10)
Fairly often	32.0 (8)	Nearly every day	4.0 (1)
Very Often	8.0 (2)		
*Question 4: In the last month, how often have you felt difficulties were piling up so high that you could not overcome them?*		*Question 2: Over the last 2 weeks, how often have you been bothered by not being able to stop or control worrying?*	
Never	4.0 (1)	Not at all	28.0 (7)
Almost never	24.0 (6)	Several days	56.0 (14)
Sometimes	40.0 (10)	More than half the days	16.0 (4)
Fairly often	24.0 (6)	Nearly every day	00.0 (0)
Very Often	8.0 (2)		

**Table A2:** Quotes from participants about impacts on psychological wellbeing.

Quote Number	Quote
Quote 1	I think that I would honestly say like my mental health, right now, is really quite good. I think that in a lot of ways […] a lot of us have like grown a lot through this and really learned to prioritize […] If you’d asked me this like March of last year, April, May of last year, it would have been a little different. I think that that was a really hard time. Like, I’ve never been somebody who has insomnia, but I would like, you know, just wake up in a cold sweat, wake up worrying, just really afraid because we had done some math based on some projections of like, if we didn’t lock down what would that look like? And like, it was really scary for our town. Like we have four ventilators at the best of times, maybe not…maybe only three actually. And we were projecting like you know 300 people would end up in the hospital […] So like, it was stuff of nightmares. Like we were just like projecting that we’d be working hundred-hour weeks and that we’d have no help, and we just didn’t know what was coming so it was just…it was really scary. (Participant 1)
Quote 2	Yeah, I mean, mental health wise, I would say, I was more stressed by the concerns or responsibilities that were mine and [what] I owned than anything else really. So, again, that sort of morphed throughout the pandemic from the very beginning when I was most worried about my daughter’s potential exposures at work, and then quickly realizing that my exposure and my long-term care residents were my biggest responsibility. That was extremely difficult. […] I think I was just exhausted by it, you know. I didn’t cry about it. I didn’t…I couldn’t get away from it. It was something that I wasn’t used to having that constant stress. You know, [I’ve had] stressful times at [my] various places of work, but I’d never had this constant, “don’t leave it” stress from, you know, last March right through to when…our residents didn’t get vaccinated until…fully vaccinated till the end of February beginning of March. So that was a long haul. (Participant 2)
Quote 3	I would say that, you know, I tend to be pretty calm, [but] I have felt more emotional recently. And you know, that maybe shows up on a zoom meeting or something like that. […] I don’t know how much of that to tribute to the pandemic. I think it’s because of not having enough physicians to do the job, so we’re all working too hard. And so then, being short of sleep. (Participant 3)
Quote 4	[I told myself] “Well, I worked every day for the past two months. Obviously, that wasn’t good enough” …so just like lots of downplaying the amount of work that I did and kind of self-deprivation that was going on as well. Now, you know, I’ve come to the realization that you know, things will eventually get better but definitely in a very dark place where it’s like, you know, what’s even the point sort of thing if, if this is…if this is the new the new way of life? (Participant 3)
Quote 5	Just because it’s been now a year and a half, and it’s not like before that year and a half, everything’s peachy right? It was a struggle to access resources prior. And then you have a pandemic that augments that exponentially. Right? And, you know, yeah. So I would say that yes, there definitely has been an effect on one’s mental health, for sure. (Participant 10)

**Table A3:** Quotes from participants about stressors affecting psychological wellbeing of rural physicians.

Stressor	Quote
Excessive Increase in Workload and Responsibilities	Being in a rural area, we are expected to cover the hospital and the emergency department as well as outpatient clinics, so there’s increased demand. Again, covering for the acute COVID patients, vaccine clinics, swabbing clinics, all of that falls onto family physicians because we are literally the only physicians in the area, so it all has to come to us. That’s probably the biggest thing…is having to cover everything and trying to pick up all the pieces of this new pandemic world. (Participant 4) All of a sudden I ended up with all of these leadership responsibilities, a lot more meetings to attend, a lot more just like mental load of like, you know, participating in not only all the planning and the kind of figuring out how we were going to do things locally, but also needing to stay up to date with the torrent, like the fire hose of information that was, you know, coming out daily in terms of like this novel virus and like what do we know about it? […] What kind of measures do we need to take to protect our staff? PPE, like you know all the protocols and treatments, just so much information to filter through. (Participant 5) And then it’s like which source do you listen to, you know? Different information coming from, you know…not hugely different, but different information coming from different sources. It was overwhelming. Like, I don’t find it as bad right now. Like I don’t get the same volume of emails, but it was easily like 15 to 20 emails a day. And it has just become like…it’s just too much to kind of keep track of. (Participant 6)
Management of Risk of Infection	I really felt like the best description that they were asking of us was that we were cannon fodder. That we were going into emergency with absolutely no backup, no hope of coming out and a high risk of getting infected. (Participant 7) It’s like watching a scary movie where you know there’s no creature that’s out, but just the anticipation is almost worse than to see it. So, I found that, you know, we would always be kind of on high alert whenever we had someone with symptoms and there would be a few situations where, you know, I would come in contact with someone that ended up being COVID positive a few days later. And of course, you know, I wore the appropriate PPE, but you’re always wondering oh you know, “Did I wash my hands right? Was there a little bit of exposure?” (Participant 8) I was coming home and stripping down in the garage and throwing my clothes directly into the washing machine, going to the shower before I would go near my kids. I still do that. (Participant 4) Like we got vaccines and then all of a sudden, they’re sent to Toronto instead. Meanwhile, everybody from Toronto was coming up here to vacation. It was just…like that made me feel 100% betrayed, you know. (Participant 9)
Management of Limited Resources in a Rural Setting	I have my N95 [now] if I need it. Like, you know, it just felt like I had some form of, like, protection, because before that it felt like we were just being like lambs to the slaughter—like just go out, work, and you can’t wear PPE because we don’t have enough. So, it was…that was terrifying. (Participant 1) When the pandemic started, we had one ventilator, which is used usually just to transport people to higher level centers. So then of course we were like well what happens if we can’t transfer them, right? Like what if the higher-level centers are full and they won’t take them? So, then we were having to train our…trying to train our nurses on how to care for a ventilated patient, which is not something that they typically do. (Participant 5) …And as someone trained in a very urban program, who is now working a 100% as a rural doctor, it feels clearer than ever, especially during the pandemic, that a lot of the people that made policies in urban centres, and a lot of people that are being trained in urban centres and practice medicine in urban centres have no idea what’s going on in rural places. (Participant 10) Remember, like everything in an urban center is augmented that much more in a rural center. Like urban centers have much higher physician per capita, right? So, there’s more redundancy. So, if someone gets sick you have…like there in a rural center, […] you’re one person away from your emerg shutting down even before COVID. (Participant 10) The irony is that we’re supposed to take care of ourselves as physicians to take care of our patients, but we’re not really allowed quote unquote to take any time off. So how do you take care of…like how do you take care of yourself when you can’t devote any time to your own self care?...There’s this weird perception of doctors where we’re supposed to be superhuman, and we can just keep on going while denying our own needs and still be excellent all the time for patients, which I find ridiculous, but that’s, I just feel like that’s kind of an expectation for physicians. (Participant 11)
Impact on Familial, Professional and Public Relationships	We basically spent four months not touching, not hugging, not sleeping in the same bed. That was stressful. It wasn’t so much around the decision making, but really around protecting capacity for the local health care system, so that we didn’t lose two docs at the same time. (Participant 12) Like you’re here either at work working or you’re at home parenting. Whereas before there used to be little pockets of time before the kids came home from school or, you know, before you know something else happened. Like so those pockets of time or windows of opportunity for self-care just evaporated and they’re still largely gone. […] You just don’t have those shared opportunities to kind of indulge in our relationship as a couple. […] I’d sort of say, my partner doesn’t feel as fun, but not to any fault of hers or mine. It just feels like, again, it’s all work all the time so… (Participant 11) I was pretty disillusioned with the public’s behavior at large. I found it very difficult. I’m going to have trouble, integrating with some of my neighbors who basically chose to ignore every public health guideline that has been, you know, put out to the public. So, I’m becoming less judgmental of them as time goes by, but it’s going to be hard to sort of reintegrate to those. (Participant 2) But I think media, especially social media has been extremely hard on physicians. […] And this gradual transition from being the hero savior who was, you know, the well-loved by the community to being the bad guy who was continuing to try and force public health and Ontario health recommendations for isolation. Feeling that at some point, a portion of the population, not all and probably not the majority, turned against us. (Participant 13)

**Table A4:** Quotes from participants about coping with the COVID-19 pandemic.

Stressor	Quote
Use of Coping Strategies	Personally, I think that, you know, because there were a lot of work responsibilities and meetings and other kind of work-related and patient care-related events, you know what, I would say that it definitely has eaten into my hobbies and my extracurricular outside of medicine interests, you know. Some of that was because you couldn’t do those interests because they weren’t, you know, available to do because of public health restrictions, you know. But often because of clinical workload. (Participant 10) My life [outside of work] hasn’t changed quite that dramatically in a rural place because there is more space. There’s more space, there’s more outdoor space, people are more used to doing outdoor things, and like generally have larger homes and patios that you can go to socialize at outdoors. (Participant 14)
Experience Accessing Physician Wellness Resources	In my mind it’s [accessing physician wellness services] something that would take even more time. I already have a busy life, which would invariably come either from my sleep or my family life. So, I just have no more time to give, including to myself. (Participant 4) You can kind of imagine sometimes in a smaller community, you might have access to in-person mental health resources but oftentimes you may know the counselor, right? And there may be a bit of familiarity with that relationship, so you may not necessarily want to discuss certain issues with that individual. (Participant 10) The other thing too I sometimes think about is if I reach out and do this and they’re concerned about me, is there any repercussions especially like on insurance? […] You know, especially for a younger physician who’s still, you know, trying to improve their insurance, you know their disability or whatever, would that have an impact, because if you say no on that questionnaire for “Have you seen a therapist in the last year?” and you lie and they find out somehow that you did and there’s reports about it or if it was copied to your family physician, that would have negative repercussions. So, I just felt there’s a barrier there, and I never felt like I was bad enough to need it. (Participant 15)
